# Multiparametric Ultrasound for Characterisation of Testicular Lesions: Beyond Orchiectomy

**DOI:** 10.3390/diagnostics16162501

**Published:** 2026-08-07

**Authors:** Michele Bertolotto, Irene Campo, Rosaria Perrone, Alberto Zucconi, Andrea Piasentin, Roberto Stramare

**Affiliations:** 1 Unit of Training & Research in Vascular & Multiparametric US, Clinical Department for Medical, Surgical and Health Sciences, University of Trieste, Ospedale di Cattinara, Strada di Fiume 447, 34149 Trieste, Italy; irenecampo11@gmail.com; 2 Department of Radiology, University of Trieste, Ospedale di Cattinara, Strada di Fiume 447, 34149 Trieste, Italy; rosaria.perrone@asugi.sanita.fvg.it (R.P.); alberto.zucconi@studenti.units.it (A.Z.); 3 Department of Urology, University of Trieste, Ospedale di Cattinara, Strada di Fiume 447, 34149 Trieste, Italy; andrea.piasentin@asugi.sanita.fvg.it; 4 Dipartimento di Scienze Cardio-Toraco-Vascolari e Sanità Pubblica, Università di Padova, Via Giustinani, 2, 35128 Padova, Italy; roberto.stramare@unipd.it

**Keywords:** testicular lesions, testicular incidentaloma, multiparametric ultrasound, testis-sparing surgery, active surveillance, orchiectomy, testicular cancer, postoperative testis

## Abstract

The increasing detection of small, non-palpable testicular lesions has challenged the traditional paradigm that every solid intratesticular mass should be managed by radical orchiectomy. Many incidental lesions, particularly in infertile men and in patients with negative tumour markers, are benign or non-neoplastic, making overtreatment a clinically relevant concern. In this setting, ultrasound has become central to a more conservative and individualised diagnostic strategy. By combining high-resolution grey-scale imaging with Doppler, microvascular imaging, CEUS and elastography, multiparametric ultrasound provides complementary information on lesion morphology, vascularity, stiffness and interval change. When integrated with clinical presentation, tumour markers, fertility status, syndromic background and patient history, these features can help characterise lesions and estimate the likelihood of malignancy in the appropriate clinical context. Although imaging cannot replace histology, it can refine pre-test probability, support active surveillance in selected low-risk lesions, guide testis-sparing surgery, and improve assessment of the operated testis. This review examines the role of multiparametric ultrasound in characterisation of focal testicular lesions across different clinical settings, including incidentalomas, acute scrotal pain, trauma, bilateral disease, heterogeneous parenchyma, genetic and endocrine syndromes, and the postoperative testis. A structured, context-sensitive imaging approach may help reduce unnecessary orchiectomy while preserving timely detection of malignant, persistent, recurrent or metachronous disease.

## 1. Introduction

According to the most recent guidelines of the European Association of Urology (EAU) and of the American Urological Association (AUA) [1,2], any solid mass in the testis identified by physical exam or imaging should be managed as a malignant neoplasm until proven otherwise. Orchidectomy remains the treatment of choice in patients with a normal contralateral testis. Testis-sparing surgery (TSS), combined with intraoperative frozen section examination, may be considered in highly selected patients with indeterminate small lesions and a high likelihood of benignity, particularly when preservation of gonadal function is desired. Active surveillance is currently not recommended by EAU guidelines, while it is considered an option in AUA guidelines for non-palpable, small (<10 mm) lesions in the absence of elevated serum tumour markers or evidence of metastatic disease [2]. In fact, malignant tumours may occur in nodules of any size [3], but unlike palpable masses, most small lesions incidentally identified in otherwise healthy men with negative tumour markers, as well as in those undergoing evaluation for infertility, are benign neoplasms and non-neoplastic lesions [4,5,6], rendering orchidectomy an inappropriately aggressive treatment in many cases, with potential adverse hormonal, reproductive and psychological consequences [7,8].

Clinical urological practice is progressively evolving towards a more conservative approach, with increasing use of testis-sparing surgery and serial US monitoring, particularly for very small lesions, in an effort to reduce overtreatment while preserving oncological safety and effectiveness. As radical orchiectomy is no longer regarded as the only management option for small non-palpable testicular lesions, the role of imaging has become increasingly important. Although imaging does not yet allow fully reliable lesion characterisation, a more educated multiparametric assessment can improve diagnostic confidence and support clinical decision-making [9]. This is essential for selecting the most appropriate management strategy, monitoring lesions managed conservatively, and planning follow-up after testis-sparing surgery.

## 2. Materials and Methods

This is a narrative, clinically oriented review. The PubMed/MEDLINE database was searched using combinations of the terms “testicular incidentaloma”, “testicular mass”, “multiparametric ultrasound”, “colour Doppler”, “microvascular imaging”, “contrast-enhanced ultrasound”, “elastography”, “testis-sparing surgery” and “active surveillance”, with no restriction on publication date but with emphasis on literature published within the last 15 years. Reference lists of retrieved articles, relevant systematic reviews and current society guidelines were hand-searched for additional relevant studies.

Where systematic reviews or meta-analyses addressing a specific clinical question were available, these were prioritised as the primary source of quantitative estimates; original cohort studies and case series were used to supplement or contextualise these estimates, and were relied upon as the principal evidence source for topics in which systematic-review-level evidence was lacking.

## 3. Clinical and Imaging Assessment of Testicular Lesions

Overall, testicular lesion characterisation remains challenging because ultrasonographic findings are often non-specific and lesion behaviour varies according to both pathological type and clinical setting. For this reason, no lesion should be interpreted on imaging alone, and a structured assessment requires careful integration of clinical history, age, ethnicity, geographical background, laboratory data, fertility status and imaging features. Bilateral disease and synchronous or metachronous lesions represent a distinct diagnostic context, as they may reflect germ cell neoplasia, lymphoma, adrenal rest tissue, Leydig cell tumours or other benign conditions, and therefore require tailored interpretation. Patient-related factors are also relevant: age strongly influences the likelihood of different tumour types, ethnic differences have been reported in the incidence of testicular germ cell tumours, with lower rates in African descent [10,11,12], although incidence in this group appears to be increasing, and the prevalence of specific lesions may vary across countries and clinical populations. Clinical presentation and serum tumour markers remain central to risk assessment, as do fertility status and associated endocrine abnormalities, since infertility is linked not only to germ cell neoplasia but also to a higher frequency of incidental and non-neoplastic lesions [1,2]. Within this framework, the testicular incidentaloma has emerged as a particularly important and increasingly common scenario.

Although lesion characterisation remains imperfect, careful grayscale ultrasound analysis may still allow an educated assessment of lesion nature [13], while a multiparametric approach may further refine assessment through the evaluation of vascularity and stiffness [9,14,15,16]. Additional complexities arise when lesions are detected during investigation of non-traumatic acute scrotal pain or after trauma, settings in which benign and non-neoplastic causes are particularly frequent. Genetic syndromes represent another important context because they may predispose to specific neoplastic or non-neoplastic lesions. Finally, the postoperative scrotum deserves separate consideration, as focal abnormalities after testis-sparing surgery or surgical sperm retrieval may represent postoperative change, residual disease, recurrence or benign non-neoplastic conditions. The following sections address each of these scenarios in greater detail.

## 4. Demographic and Clinical Context

**Figure 1:**
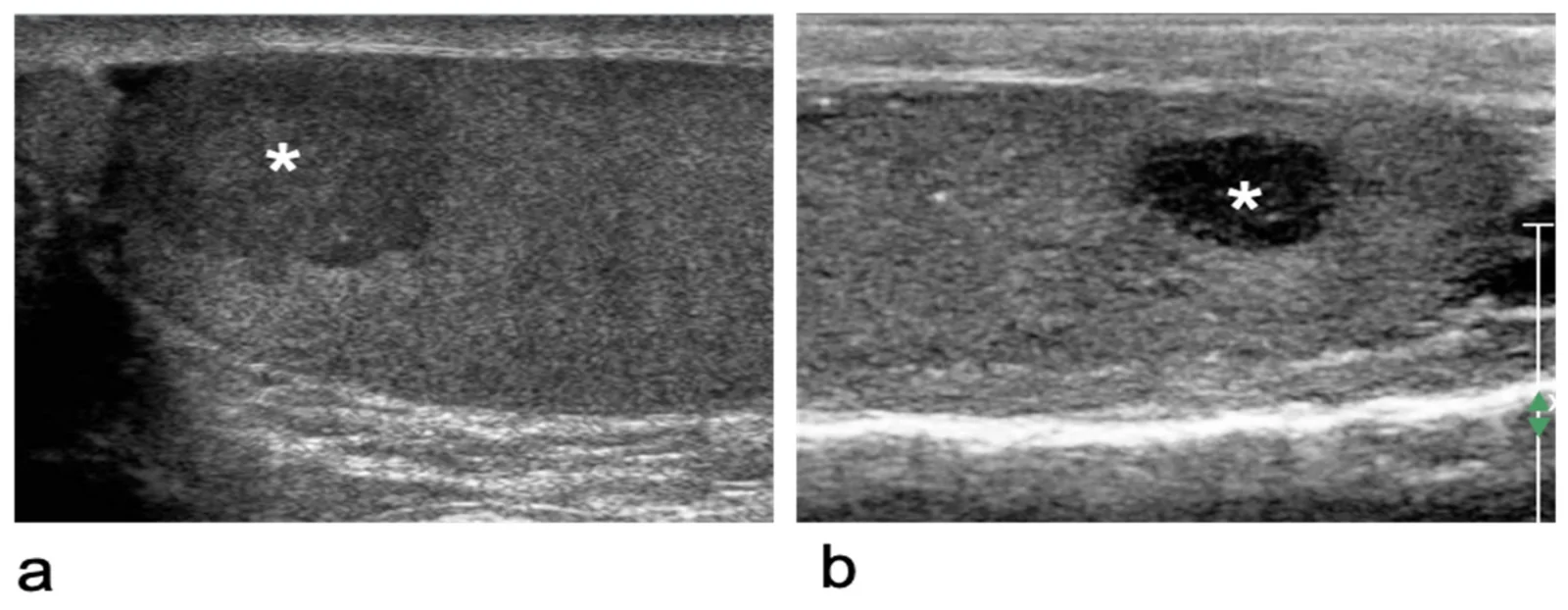
Small testicular tumours (asterisks) presenting as palpable lesions in (**a**) a 23-year-old man and (**b**) a 40-year-old man. At surgery, the lesions proved to be a non-seminomatous germ cell tumour and a pure seminoma, respectively.

Characterisation of focal testicular lesions should incorporate age, ethnicity, patient history and geographical background, as these variables influence the pre-test probability of malignancy. Testicular germ cell tumours (TGCTs) predominantly affect adolescents and young adults [17,18]. Non-seminomatous tumours tend to occur at a younger age, typically in the third decade of life, whereas seminomas typically present at a later age than non-seminomatous tumours, with a peak incidence around 35–39 years (Figure 1) [17].

Lymphoma should be particularly considered in older patients, as it represents the most common testicular malignancy in men over 60 years of age [19,20]. Accordingly, a small intratesticular lesion in a man in his twenties or thirties carries a different oncological significance from a similar finding in childhood or later life, when prepubertal-type tumours, sex cord-stromal tumours, benign lesions and, in older patients, lymphoma become relatively more relevant differential diagnoses [1].

Ethnic and geographical differences are also clinically relevant. Contemporary epidemiological studies confirm substantial international variation in TGCT incidence, with the highest rates observed in Northern and Western Europe, North America and Oceania, and the lowest in Africa and much of Asia [18,21,22]. In the United States, incidence has historically been highest among men of European descent and lowest among men of African descent, although recent data indicate a marked increase among Hispanic populations [11]. These patterns suggest a complex interplay between genetic susceptibility and environmental factors [17,22].

**Figure 2:**
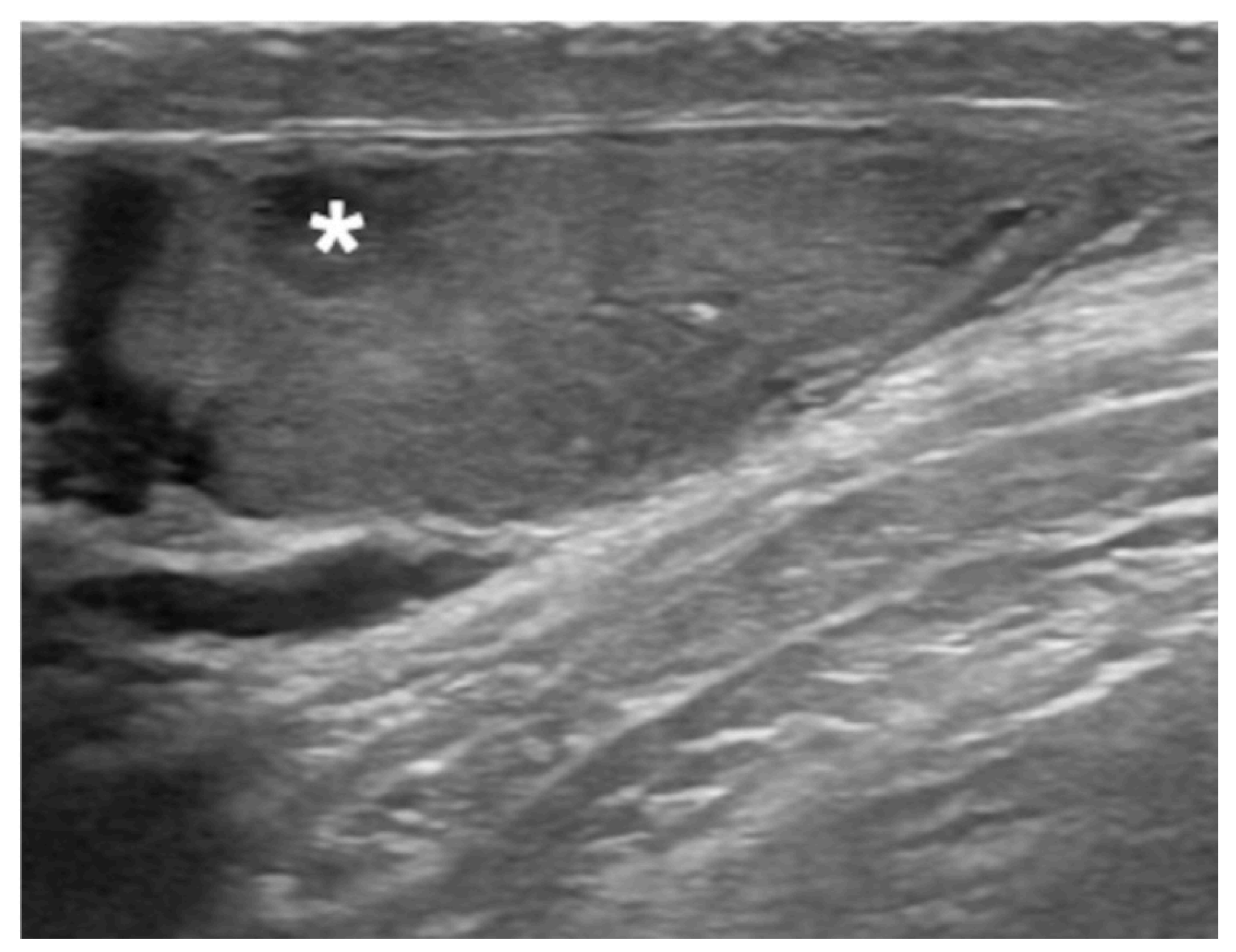
Small, non-palpable testicular lesion (asterisk) incidentally identified at ultrasound in a 45-year-old azoospermic man with bilateral testicular hypotrophy, investigated during fertility work-up. The lesion proved to be a seminoma at surgery.

**Figure 3:**
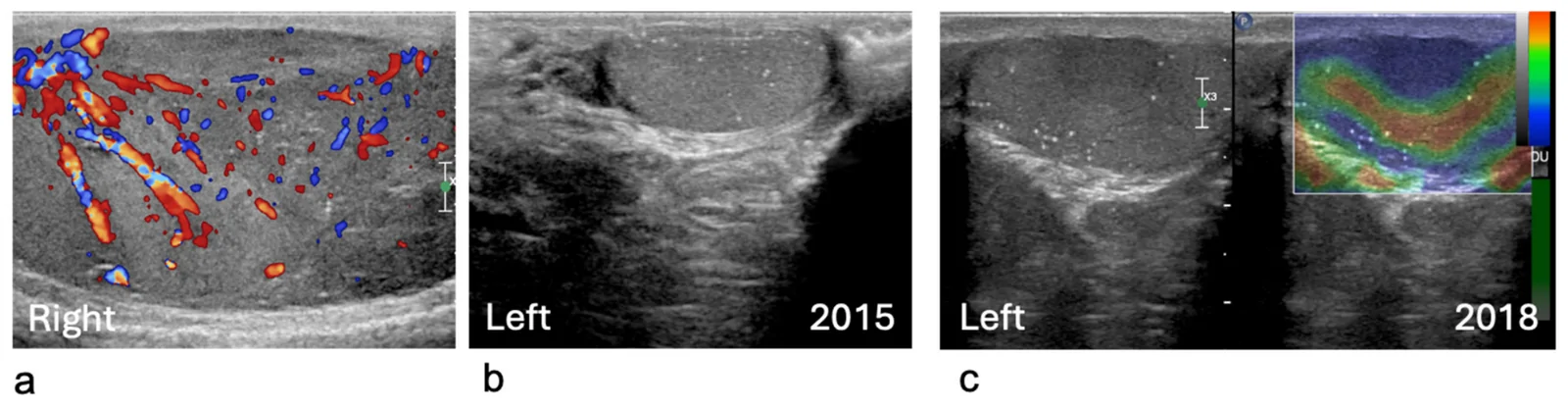
A 29-year-old infertile man presenting with swelling of the right testis and a family history of testicular cancer, with his father having had a seminoma. (**a**) Grey-scale ultrasound shows a large solid lesion causing subtotal involvement of the right testis. (**b**) The contralateral testis is small and shows testicular microlithiasis, with no focal lesion. Right orchiectomy was performed, and histology revealed a typical seminoma. (**c**) Three years later, during follow-up, ultrasound detected a non-palpable focal lesion in the remaining left testis, which showed increased stiffness on elastographic imaging. Surgery was performed, and histology again revealed a typical seminoma.

Patient history further refines risk assessment. Established risk factors for TGCT include cryptorchidism, impaired spermatogenesis or infertility (Figure 2), a personal or family history of testicular cancer, contralateral TGCT (Figure 3), and disorders of sex development/testicular dysgenesis [1,17].

**Figure 4:**
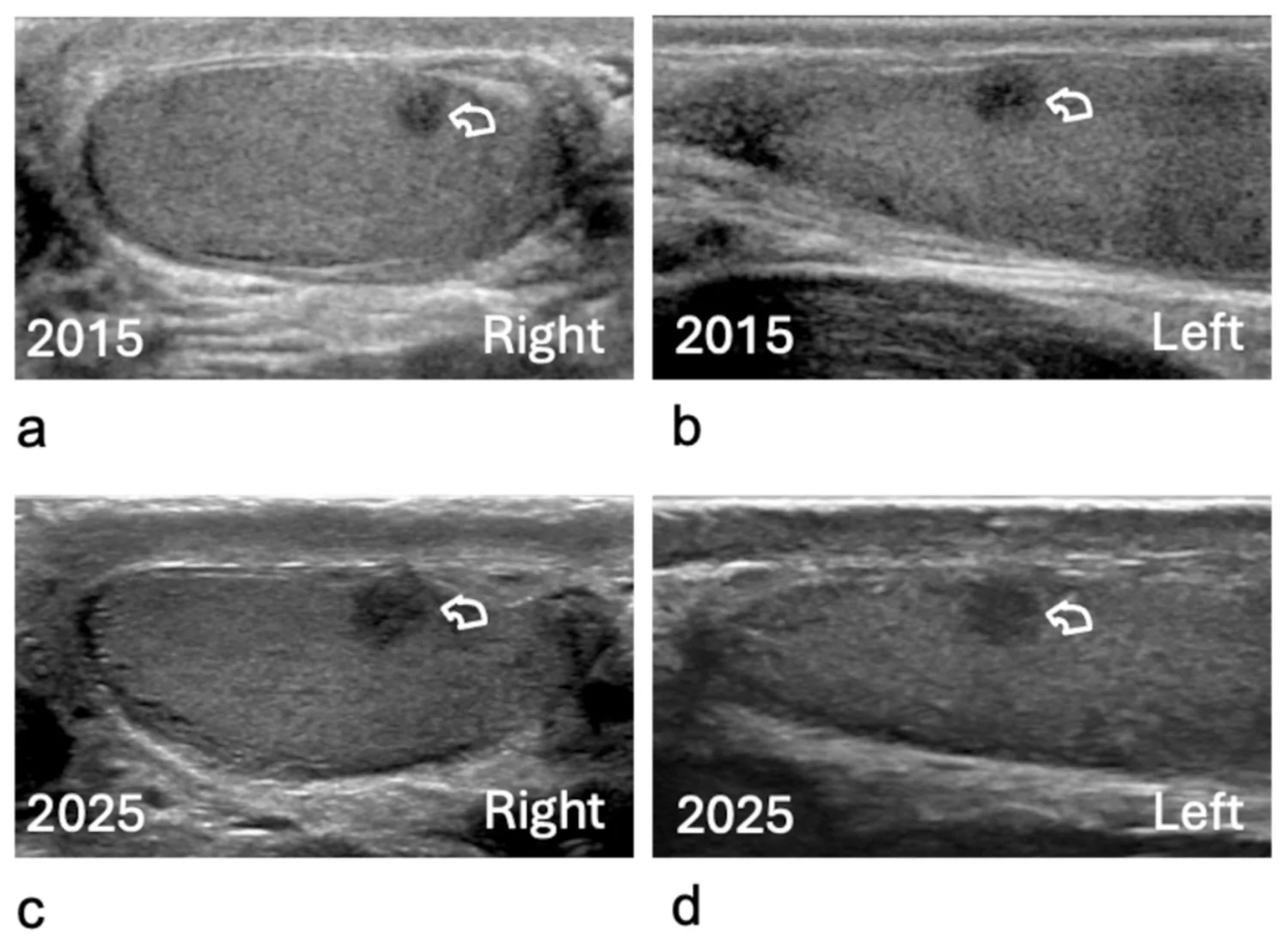
A 40-year-old infertile man. (**a**,**b**) Ultrasound shows small testes with two small, non-palpable hypoechoic focal lesions, measuring 3 mm, in the right (**a**) and left (**b**) testis, respectively (curved arrows). The patient was managed with imaging follow-up. (**c**,**d**) Follow-up examination obtained 10 years later in the same patient shows only a minimal increase in lesion size (curved arrows) to 4 mm, supporting their benign nature.

Notably, while male infertility may itself be an independent risk factor for testicular cancer [23], particularly when associated with testicular microlithiasis [24], incidental testicular lesions are more commonly detected in infertile men, in whom the likelihood of malignancy appears to be particularly low [25,26,27,28,29,30]. As surgery may further impair an already compromised reproductive potential, ultrasound surveillance appears especially appropriate in this setting, particularly for lesions < 5 mm, with negative tumour markers and no clearly suspicious ultrasound features (Figure 4) [4].

## 5. Clinical Presentation and Tumour Markers

Classical clinical indicators of malignancy include a painless testicular mass, progressive enlargement and, less commonly, associated symptoms such as heaviness or discomfort. These features, however, are usually absent in non-palpable incidentalomas, a setting in which testis-sparing surgery and active surveillance may be appropriate management options [1,31]. Evaluation of serum tumour markers plays a central role in the assessment of focal testicular lesions, although its discriminative power is limited in small, incidentally detected nodules [1,2,32,33]. Indeed, conventional markers are elevated in only approximately 50% of germ cell tumours, while non-germ-cell testicular tumours generally do not express them. Nevertheless, marker positivity substantially increases the estimated risk of malignancy: even a very small intratesticular lesion associated with elevated tumour markers is more likely to represent a germ cell neoplasm, thereby favouring a surgical rather than a conservative approach [1,27,34,35]. Elevated alpha-fetoprotein (AFP) is strongly associated with non-seminomatous germ cell tumours, whereas beta-human chorionic gonadotropin (β-hCG) may be increased in both non-seminomatous tumours and a subset of seminomas; lactate dehydrogenase (LDH) is less specific and mainly reflects tumour burden [1,32]. Circulating microRNA-371a-3p represents a highly promising novel biomarker, being detectable in approximately 90% of germ cell tumours, with the exception of teratoma [33,36]. Although it has not yet entered routine clinical practice, it is likely to improve and refine the assessment of testicular lesions and may become a future standard biomarker for germ cell tumours.

## 6. Lesion Size, Echogenicity and Echotexture

Reported estimates of the proportion of benign tumours and non-neoplastic lesions among testicular incidentalomas < 2 cm vary with study design. A two-centre retrospective service evaluation of 81 patients with lesions < 10 mm found that 56 of 81 lesions (69.1%; 95% CI 58.4–78.1%) were benign or non-neoplastic [37], while a narrative synthesis of testis-sparing surgery series reported a broadly comparable range of approximately 65–72% [38]; neither of these sources constitutes a formal meta-analysis with pooled confidence intervals.

Lesion size is one of the most useful predictors of histology: the smaller the lesion, the greater the likelihood of benignity. This association was confirmed by a comprehensive analysis of 641 surgically treated testicular neoplasms, in which the median diameter was 10 mm for benign tumours, compared with 30 mm for seminomas, 35 mm for non-seminomas and 53 mm for other malignant tumours (*p* < 0.0001). Benign histology was found in 50.6% of lesions measuring ≤ 10 mm, but in only 6.3% of those measuring > 10 mm. Receiver operating characteristic analysis yielded an area under the curve of 0.891, with a 16 mm cut-off providing 81.5% sensitivity and 81.0% specificity for distinguishing benign from malignant tumours [39]. A systematic review pooling data from 74 primary studies (1348 lesions overall, with individual lesion-level outcome data available for 408) estimated that the probability of malignancy increases by approximately 9.9% for each additional millimetre in diameter; virtually all lesions < 3 mm were benign, 86.6% of lesions < 5 mm were benign, and only 38.14% of lesions > 10 mm were benign [40]. Owing to the retrospective and heterogeneous nature of the contributing primary studies, this systematic review did not report formal confidence intervals around these pooled figures, which should therefore be interpreted as descriptive estimates rather than precision-weighted meta-analytic values. Nevertheless, the variability of the proposed size thresholds indicates that lesion diameter should not be used as an isolated criterion for clinical decision-making.

**Figure 5:**
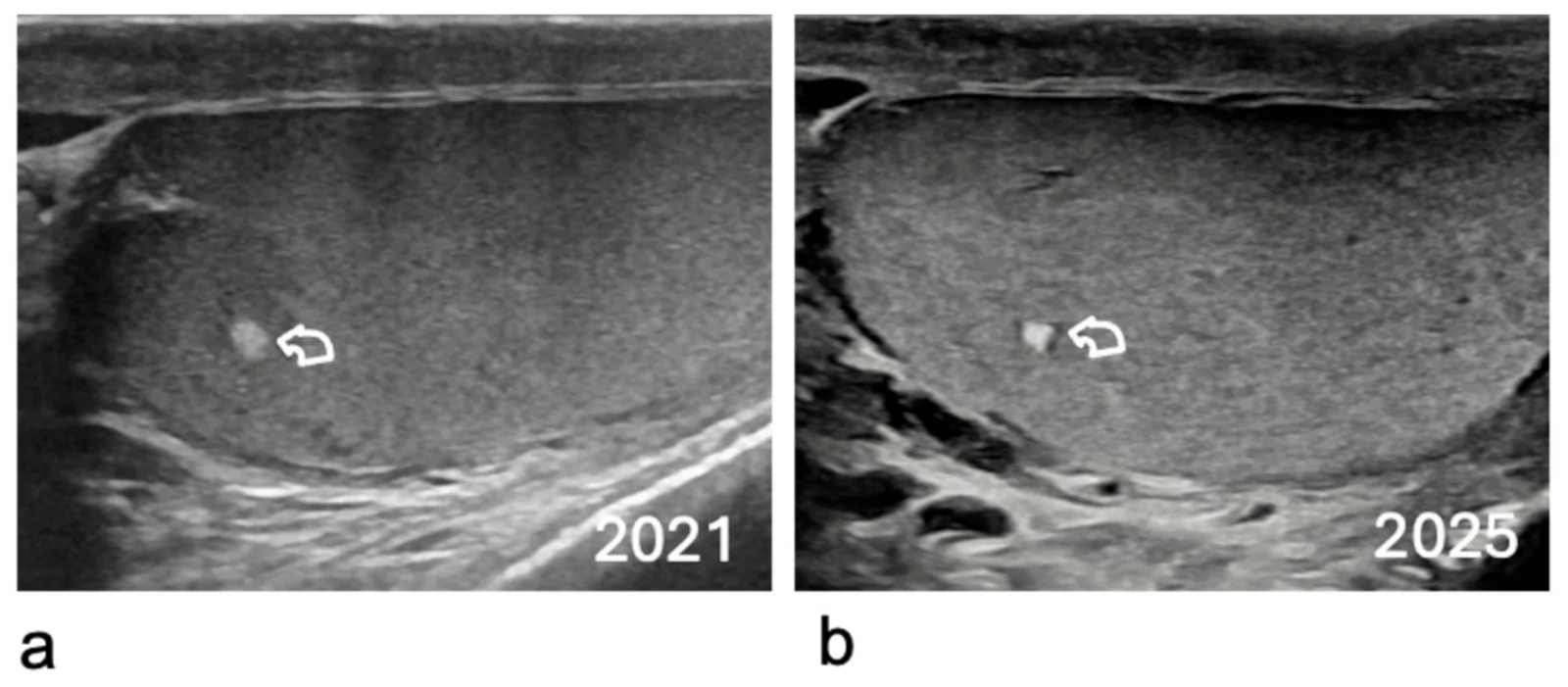
A 33-year-old man underwent ultrasonography for mild discomfort in the left testis. (**a**) A small hyperechoic lesion measuring 3 mm was detected incidentally (curved arrow). (**b**) Four-year follow-up examination shows no change in the size or appearance of the lesion (curved arrow).

Echogenicity alone has limited discriminatory value. Hyperechoic lesions are usually benign (Figure 5) [40,41] but most incidentalomas < 2 cm are homogeneously hypoechoic, irrespective of histology [40]. Seminomas largely predominate among malignant small incidentalomas and typically appear on grey-scale ultrasound as solid, well-defined, homogeneous hypoechoic nodules [40,42,43], whereas heterogeneous lesions include a broader spectrum of benign tumours and non-neoplastic lesions [37,40].

Echotexture should also be interpreted cautiously. Although one meta-analysis reported lower odds of malignancy for heterogeneous than for homogeneous small testicular lesions, this finding was based on only three studies including 51 patients overall, with no dedicated size-stratified analysis and substantial methodological heterogeneity [42]. Apart from simple cysts and typical epidermoid cysts [44], the ultrasound appearance of testicular incidentalomas is therefore usually non-specific.

## 7. Vascularity and Stiffness on Multiparametric Ultrasound

**Figure 6:**
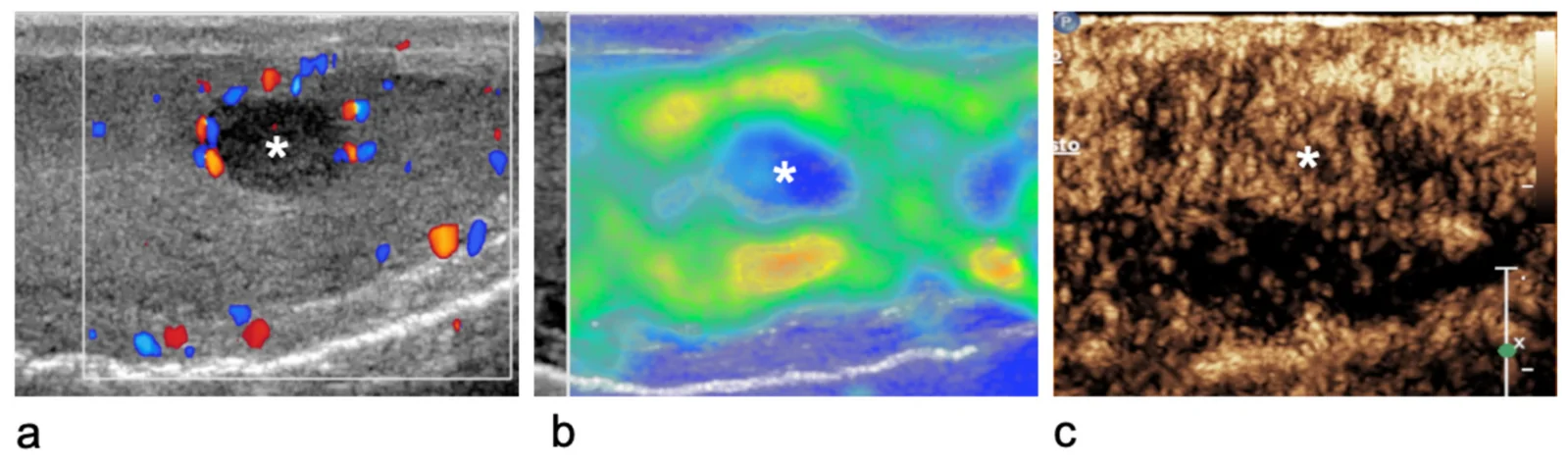
A 40-year-old man underwent ultrasonography after self-palpation of a firm lesion in the right testis. (**a**) Grey-scale and colour Doppler ultrasound show a small hypoechoic lesion (asterisk) with no apparent vascularity on colour Doppler imaging. (**b**) On elastography, the lesion (asterisk) appears stiff, in keeping with the palpable finding. (**c**) CEUS demonstrates hypervascularity within the lesion (asterisk). At surgery, the lesion proved to be a pure seminoma.

**Figure 7:**
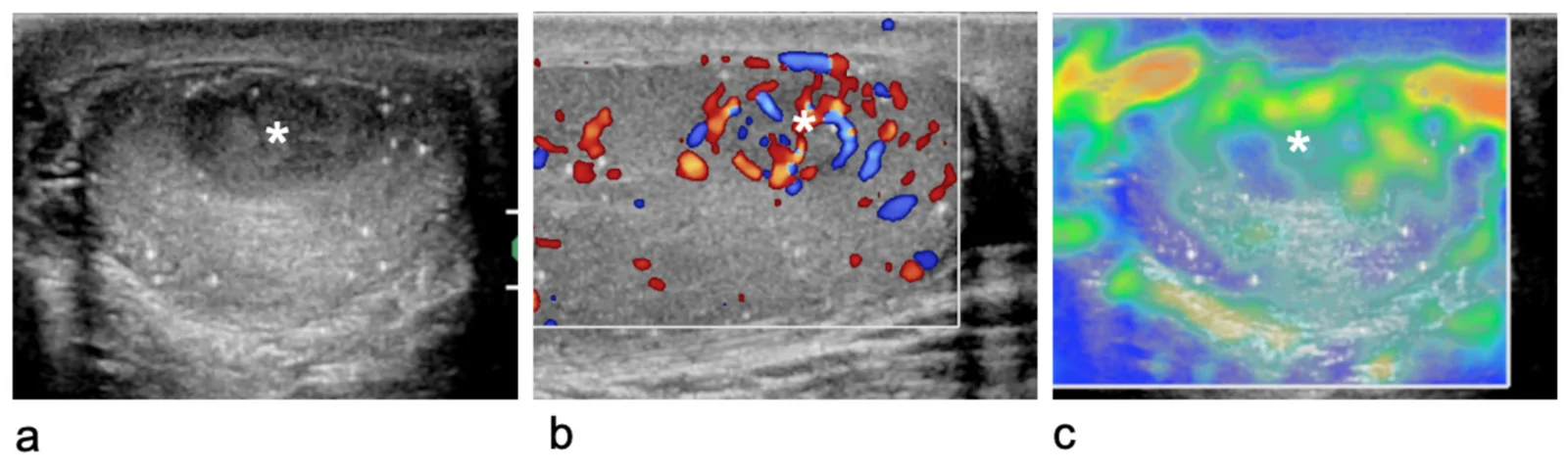
Small, non-palpable testicular lesion (asterisk) incidentally detected at ultrasound in a 25-year-old man investigated during fertility work-up. (**a**) Grey-scale ultrasound shows a heterogeneous left testis with microlithiasis and a hypoechoic lesion. The lesion is hypervascular on colour Doppler imaging (**b**) and predominantly soft on elastography (**c**). At surgery, the lesion proved to be a seminoma.

Several studies have identified imaging features associated with a higher likelihood of benignity, including absent vascularity on colour Doppler ultrasound and a soft appearance on elastography, although substantial overlap with malignant lesions remains (Figure 6 and Figure 7) [45,46,47].

**Figure 8:**
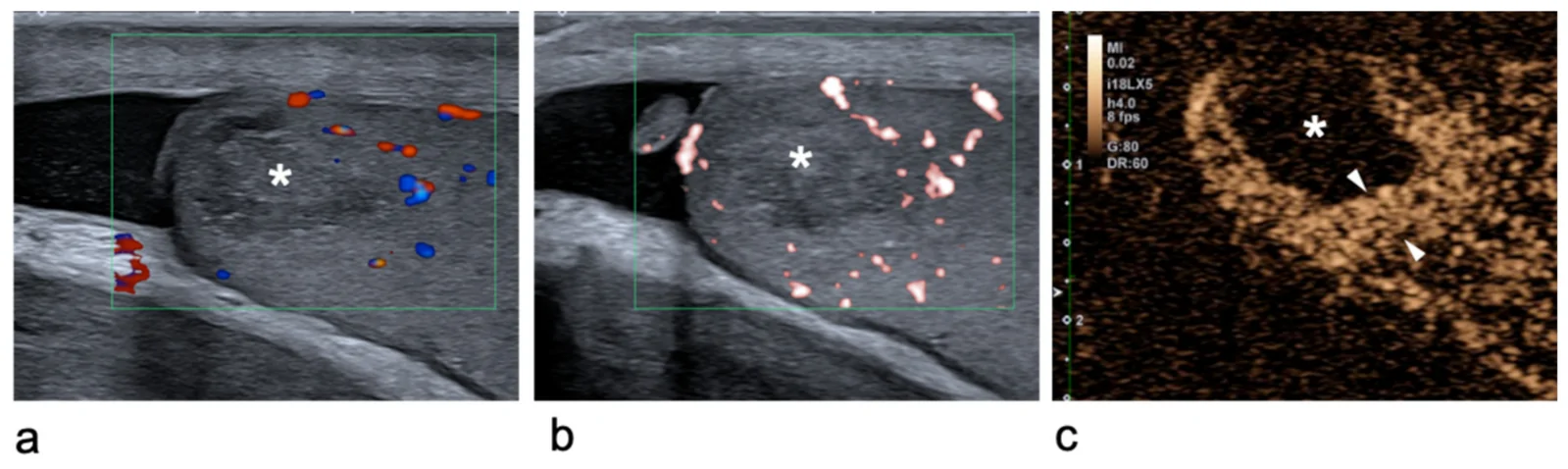
A 49-year-old man underwent ultrasonography following an episode of pain in the left testis one week earlier. He was asymptomatic at the time of examination. (**a**,**b**) Ultrasound shows a heterogeneously hypoechoic lesion (asterisk) at the upper pole of the left testis, with no vascularity on colour Doppler (**a**) or microvascular imaging (**b**). The absence of vascularity (asterisk) is confirmed on CEUS (**c**), which also demonstrates a rim of perilesional enhancement (arrowheads), suggestive of subacute segmental testicular infarction.

Lesions showing no vascularity at contrast-enhanced ultrasound (CEUS) are almost invariably benign (Figure 8), whereas vascularised lesions may be either benign or malignant [47,48,49].

**Figure 9:**
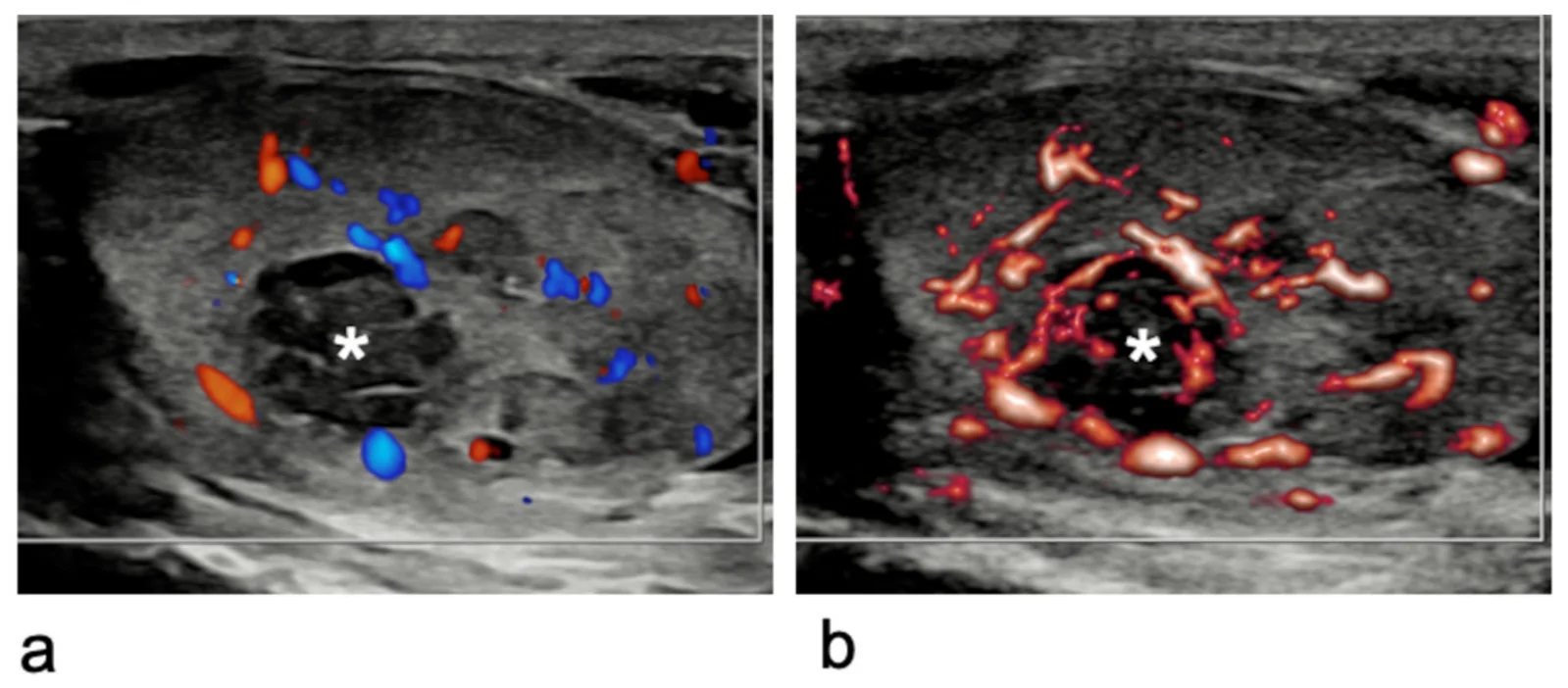
A 35-year-old man underwent ultrasonography for left testicular discomfort persisting for more than one week after minor trauma. (**a**,**b**) Ultrasound shows a hypoechoic lesion (asterisk) at the upper pole of the left testis, with no vascularity on colour Doppler imaging (**a**) but evident vascularity on microvascular imaging (**b**). At surgery, the lesion proved to be a seminoma.

The possibility of distinguishing lesions with different malignant potential on the basis of dynamic CEUS, through analysis of time–intensity curves following microbubble contrast administration, remains investigational [4,19]. Microvascular imaging (MVI) techniques improve sensitivity for slow and low-volume flow, thereby increasing the likelihood of detecting vascularity in lesions that appear avascular on conventional Doppler imaging (Figure 9) [4,9,49,50,51]. MVI is not a single, standardised technique: vendor-specific implementations rely on proprietary, non-interchangeable clutter-suppression and adaptive-filtering algorithms, and consequently differ in their sensitivity to slow flow and in their susceptibility to motion and background-noise artefacts; direct head-to-head comparisons between these platforms remain scarce [52]. Use of MVI may reduce the need for CEUS, since the demonstration of intralesional microvascularity already supports the presence of viable tissue.

However, when a lesion appears avascular on both colour Doppler and MVI, CEUS remains the reference standard for assessing vascularity [4,9,53,54]. Importantly, the use of CEUS in this setting should not be discouraged by safety concerns, as ultrasound contrast agents have an excellent safety profile and severe adverse reactions are very rare. Because microbubbles remain intravascular and are not nephrotoxic, CEUS can be used safely when clinically indicated to clarify lesion vascularity and support lesion characterisation [55].

Elastography provides complementary information by assessing tissue stiffness. Malignant tumours are, on average, stiffer than non-neoplastic lesions, but the degree of overlap is considerable, since benign tumours, fibrosis, and organised haematomas may also appear stiff [9,49,56,57,58]. Accordingly, elastography should be considered an adjunct rather than a stand-alone technique for malignancy risk stratification and interpreted in conjunction with B-mode, Doppler, MVI and CEUS findings [9].

Overall, multiparametric ultrasound improves diagnostic confidence, supports the selection of lesions suitable for surveillance or testis-sparing surgery, and may help reduce unnecessary radical orchiectomy without compromising oncological safety [4,9,51].

## 8. Incidentalomas in the Setting of Non-Traumatic Acute Scrotal Pain

**Figure 10:**
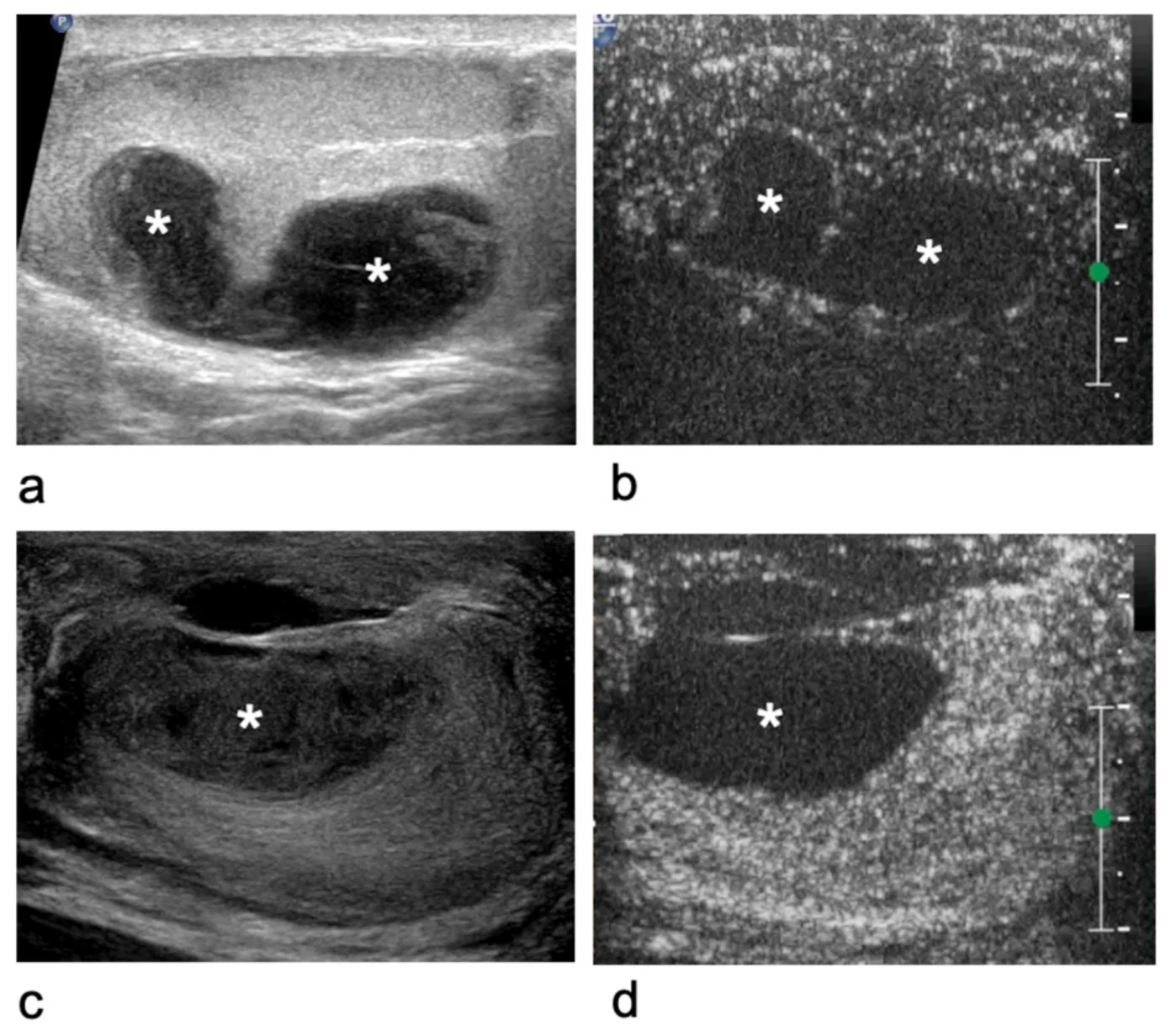
Benign lesions (asterisks) presenting clinically with acute scrotal pain and appearing avascular at CEUS. (**a**,**b**) Spontaneous intratesticular haematomas; (**c**,**d**) abscess formation.

When a testicular incidentaloma is detected in the setting of non-traumatic acute scrotal pain, the spectrum of underlying disease differs substantially from that observed in asymptomatic patients. Non-neoplastic lesions are proportionally more frequent and include segmental testicular infarction, spontaneous intratesticular haematoma, focal abscess formation, and less common vascular disorders such as vasculitis-related ischaemic lesions or venous thrombosis. These lesions are typically avascular on Doppler and CEUS (Figure 8 and Figure 10) [9,48,59,60,61].

Nevertheless, a substantial minority of patients with testicular tumours may also present with acute scrotal pain [60,62].

In this context, assessment of vascularity is central to lesion characterisation. Conventional colour Doppler ultrasound may be insufficient to demonstrate vascularity in small hypovascular lesions, whereas microvascular imaging can improve the detection of slow-flow signals, thereby reducing the need for CEUS in selected cases [9,63]. Elastography may provide ancillary information, but its role is secondary because stiffness patterns overlap considerably between infarction, haematoma, inflammation, benign tumours, and malignant lesions [9,56,64]. Overall, multiparametric ultrasound is valuable for appropriately contextualising focal lesions in the painful testis and for helping to avoid unnecessary orchiectomy [9,61,63].

## 9. Incidentalomas in the Setting of Trauma

**Figure 11:**
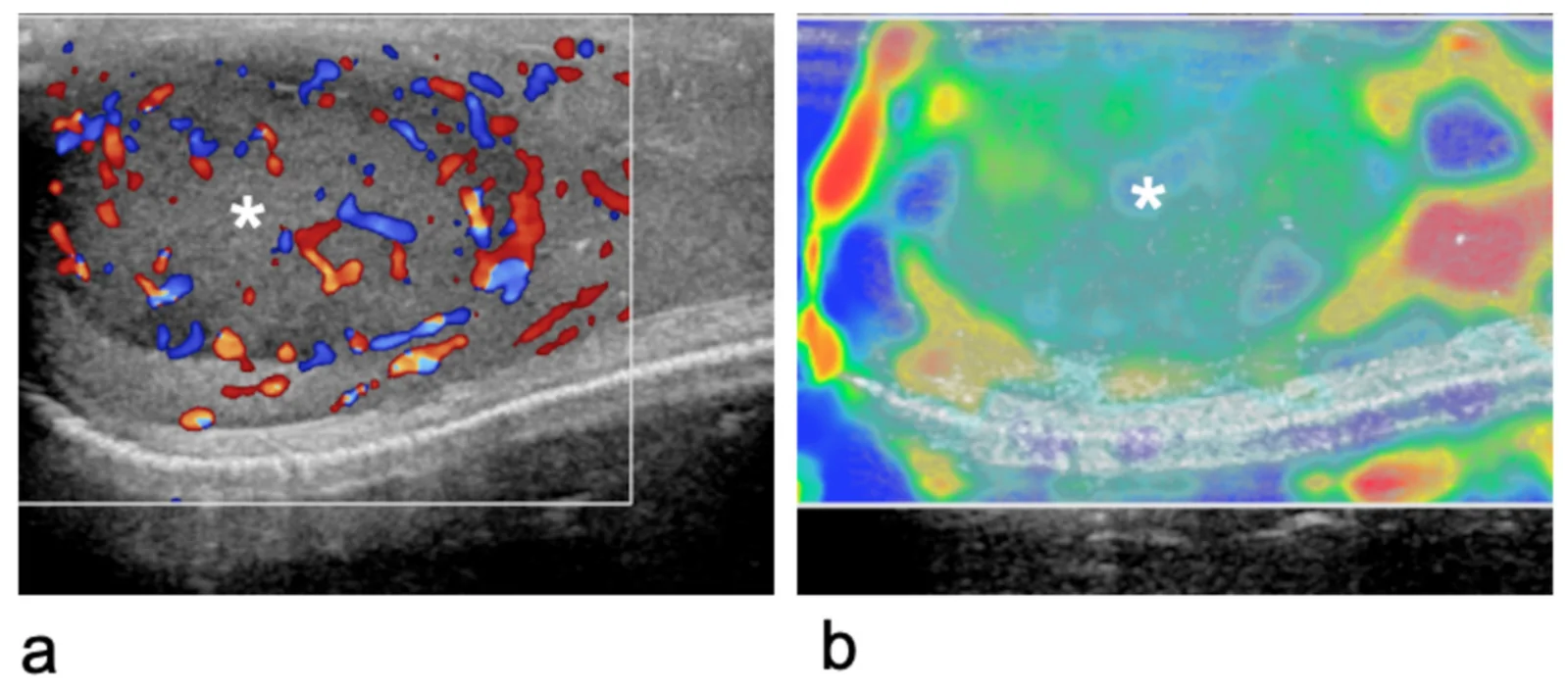
A 26-year-old man was referred for painful enlargement of the right testis that had developed after minor scrotal trauma three weeks earlier. In the preceding days, he had noticed a reduction in the size of the right hemiscrotum. He denied fever, and no palpable lesion was detected. Ultrasound shows a testicular lesion (asterisk) that is markedly vascular on colour Doppler imaging (**a**) and soft on elastography (**b**). At surgery, the lesion proved to be a seminoma with intralesional haemorrhage, likely secondary to the preceding trauma.

Incidentalomas are frequently detected during ultrasound evaluation of scrotal trauma, but their prevalence and clinical significance differ substantially from those observed in asymptomatic patients. Most focal abnormalities in this setting are non-neoplastic and reflect traumatic injury, including intratesticular haematoma, testicular rupture, focal devascularisation, contusion, and areas of infarction or necrosis [9,57,63,65]. Nevertheless, trauma may occasionally lead to the incidental detection of an underlying tumour, either because imaging is performed for pain and swelling or because haemorrhage within a neoplasm renders the lesion clinically evident (Figure 11) [9]. For this reason, the interpretation of focal lesions after trauma requires careful correlation with the clinical history and, above all, with lesion vascularity.

Colour Doppler ultrasound provides the initial evaluation, MVI increases sensitivity for slow and low-volume flow, and CEUS remains the reference standard when definitive confirmation of absent or residual perfusion is required [9,57,63,64,65,66]. The distinction between avascular and hypovascular lesions has direct implications for management, since non-enhancing lesions support conservative treatment, whereas enhancement raises concern for viable tissue, ongoing haemorrhage or, less commonly, an underlying neoplasm [9,63,66]. Short-term follow-up is also important in the differential diagnosis.

Traumatic focal lesions typically change rapidly in echotexture, shape and size, and usually decrease over time as the haematoma evolves, whereas testicular tumours remain stable or show interval growth on early follow-up [41,67]. Temporal evolution is therefore a valuable additional parameter when the initial imaging appearance is indeterminate, particularly in avascular or minimally vascular lesions managed conservatively.

## 10. Bilateral Incidentalomas

**Figure 12:**
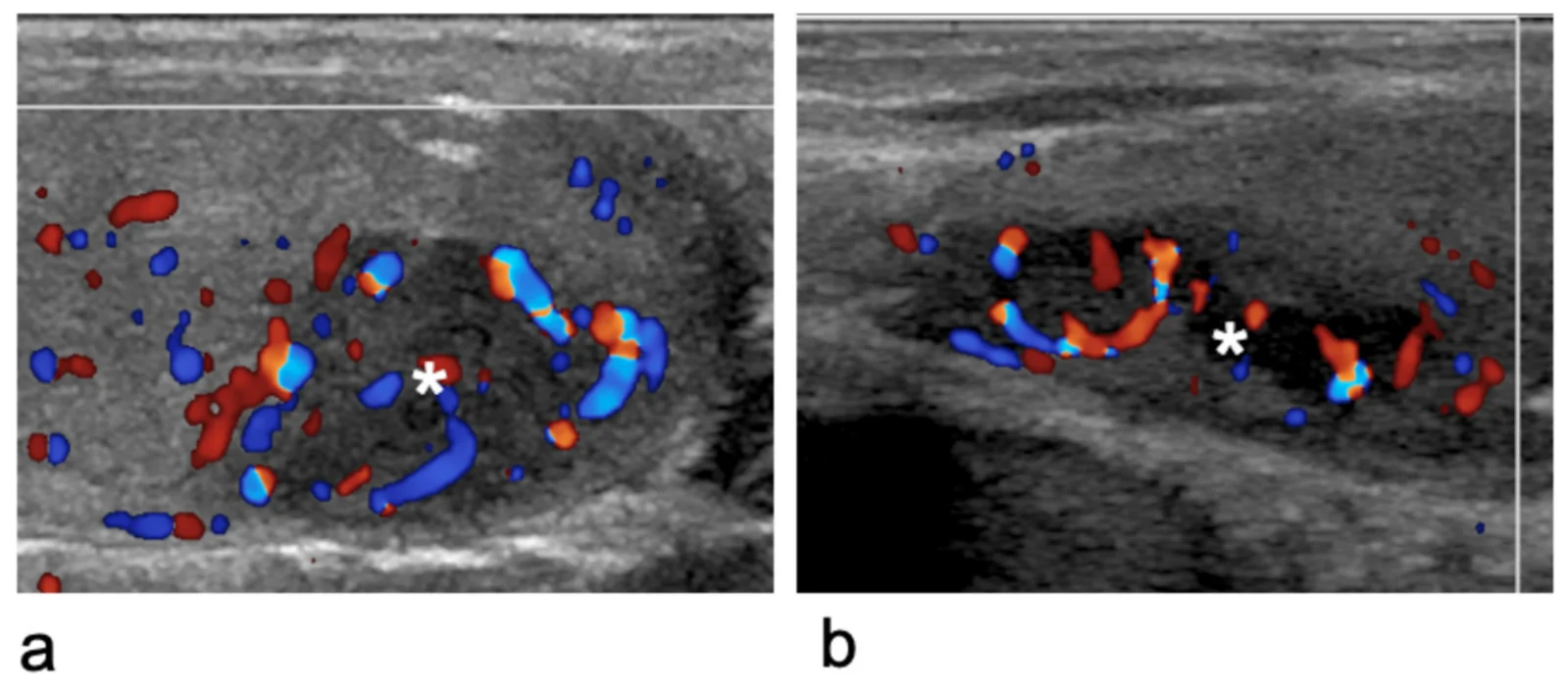
A 46-year-old man was referred for ultrasound for infertility. (**a**,**b**) Right (**a**) and left (**b**) non-palpable hypoechoic testicular lesions (asterisks) with hypervascularity were detected on ultrasound. Tumour markers, ACTH, 17-OHP and androstenedione levels were normal, making a diagnosis of testicular adrenal rest tumours unlikely. At surgery, the lesions proved to be bilateral pure synchronous seminomas.

A second primary tumour in the contralateral testis may occur in up to 5% of men with a previous testicular tumour (Figure 3 and Figure 12) [68]. Approximately 35% of these cases are synchronous and 65% metachronous [66]. Seminoma is the most frequent histology among bilateral tumours, but concordant histology is not invariable [69]. Discordant histology has been reported in approximately one third of bilateral germ cell tumours in a large systematic review [68], a proportion recently confirmed in a long-term single-centre cohort study [70]. Bilateral epidermoid cysts have also been described [71].

Current urological guidelines recognise testis-sparing surgery as a valid option in selected patients with bilateral tumours or a tumour in a solitary testis, provided that tumour volume is limited, more than 50% of testicular parenchyma can be preserved, and the patient is counselled regarding the risk of local relapse and the potential need for adjuvant treatment [1,2,35]. In this setting, ultrasound has a central role not only in detecting additional lesions but also in long-term surveillance, since metachronous tumours may arise years after treatment of the first neoplasm [1,2,35].

## 11. Heterogeneous Testicular Parenchyma

**Figure 13:**
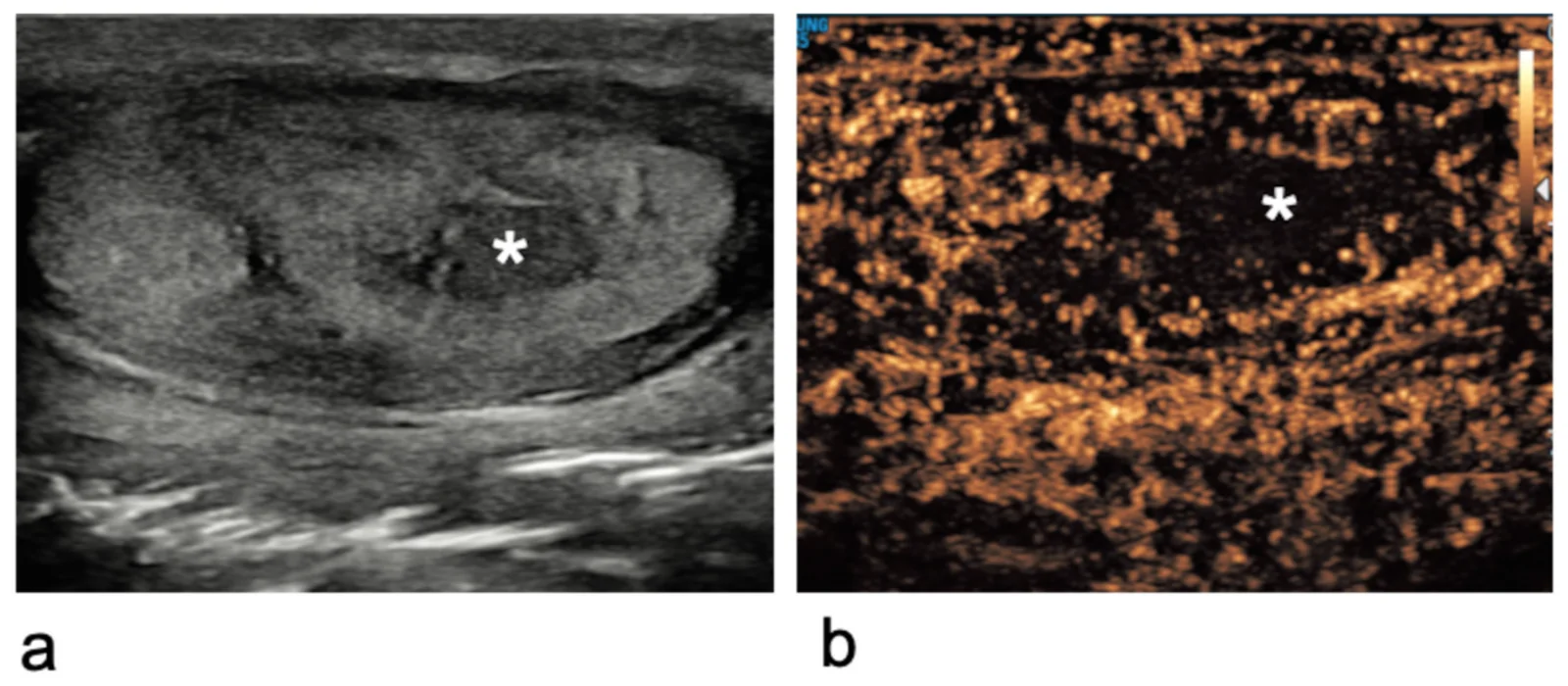
A 75-year-old man with a history of severe right epididymo-orchitis underwent ultrasonography for mild testicular discomfort. (**a**) Ultrasound showed a markedly heterogeneous right testis, with a focal hypoechoic lesion visible within it (asterisk). (**b**) CEUS demonstrated that the lesion (asterisk) was avascular, excluding an underlying neoplasm and supporting a diagnosis of post-inflammatory ischaemic changes.

After testis-sparing surgery, or when assessing the contralateral testis, heterogeneous residual or contralateral parenchyma may complicate interpretation. Heterogeneous echotexture is not synonymous with tumour and more often reflects parenchymal atrophy, fibrosis, previous injury, cryptorchidism, gonadal dysgenesis, or Klinefelter syndrome. However, it may reduce lesion conspicuity and obscure small residual, recurrent, or metachronous tumours [9]. For this reason, both the residual parenchyma after testis-sparing surgery and the contralateral testis should be carefully and systematically assessed. Multiparametric ultrasound may be useful in this setting, as it can improve the detection, conspicuity and characterisation of focal lesions developing within a heterogeneous background (Figure 13) [9].

In practical terms, heterogeneous testicular parenchyma, whether in the residual testis after testis-sparing surgery or in the contralateral testis, should not by itself prompt surgery if no discrete vascularised lesion is identified. However, it should lead to clinical correlation, tumour-marker assessment, and tailored ultrasound follow-up, particularly in patients with a history of testicular germ-cell tumour [1,9].

## 12. Genetic Syndromes

**Figure 14:**
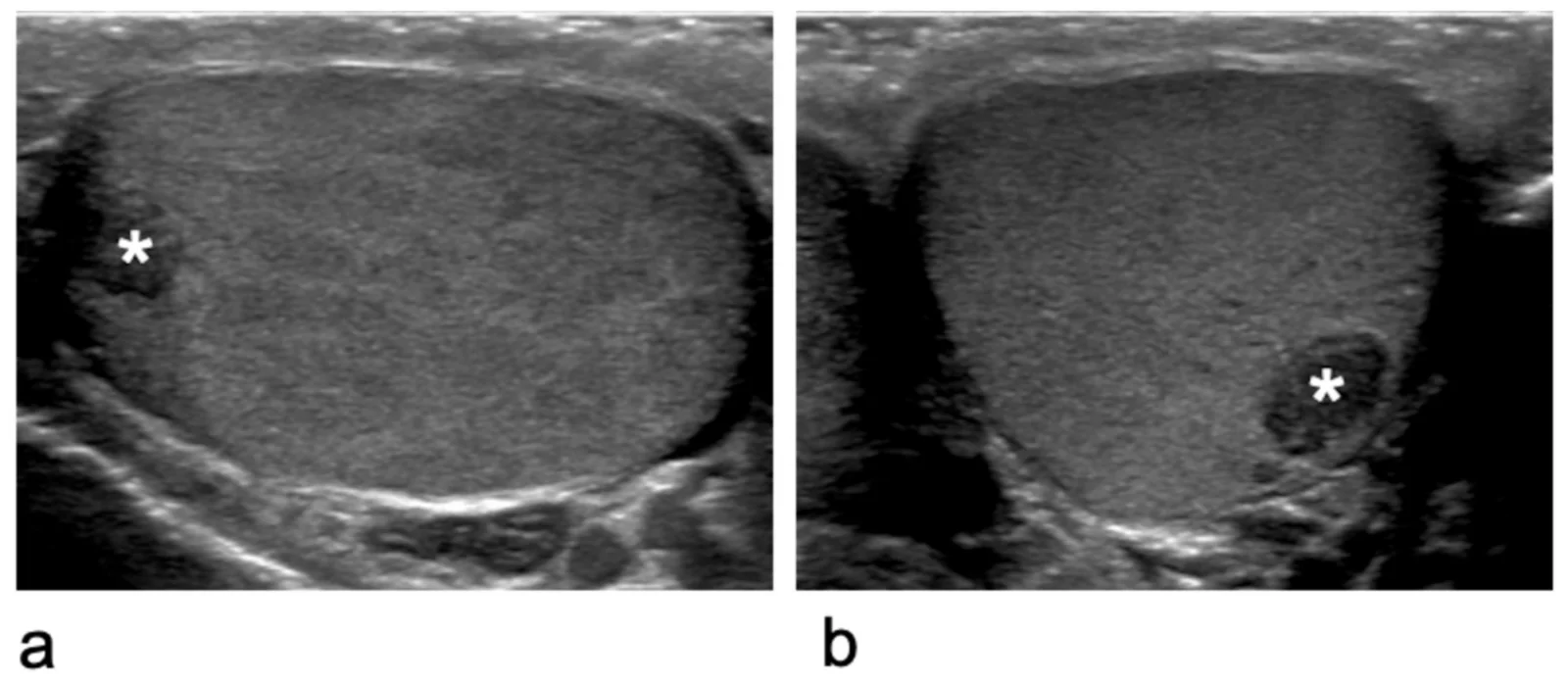
A 26-year-old man with congenital adrenal hyperplasia (CAH). Ultrasound shows bilateral testicular involvement by hypoechoic testicular adrenal rest tumours (TARTs), visible in the right (**a**) and left (**b**) testes (asterisks) near the rete testis.

In patients with genetic or endocrine syndromes, focal testicular lesions require a different diagnostic framework from that used for sporadic lesions, because the underlying condition changes both the pre-test probability of malignancy and the expected lesion spectrum. In this setting, bilateral, multifocal, or mediastinum-centred lesions are more often benign or syndrome-related than malignant. Since benign lesions predominate and the pre-test probability differs from that of the general population, a non-surgical approach is appropriate in most cases, based on clinical correlation, endocrine assessment, and tailored imaging follow-up [9,51,72,73]. The best recognised example is congenital adrenal hyperplasia (CAH), in which testicular adrenal rest tumours (TARTs) are common and typically appear as bilateral, often lobulated hypoechoic lesions near the mediastinum testis (Figure 14) [9,51,72].

**Figure 15:**
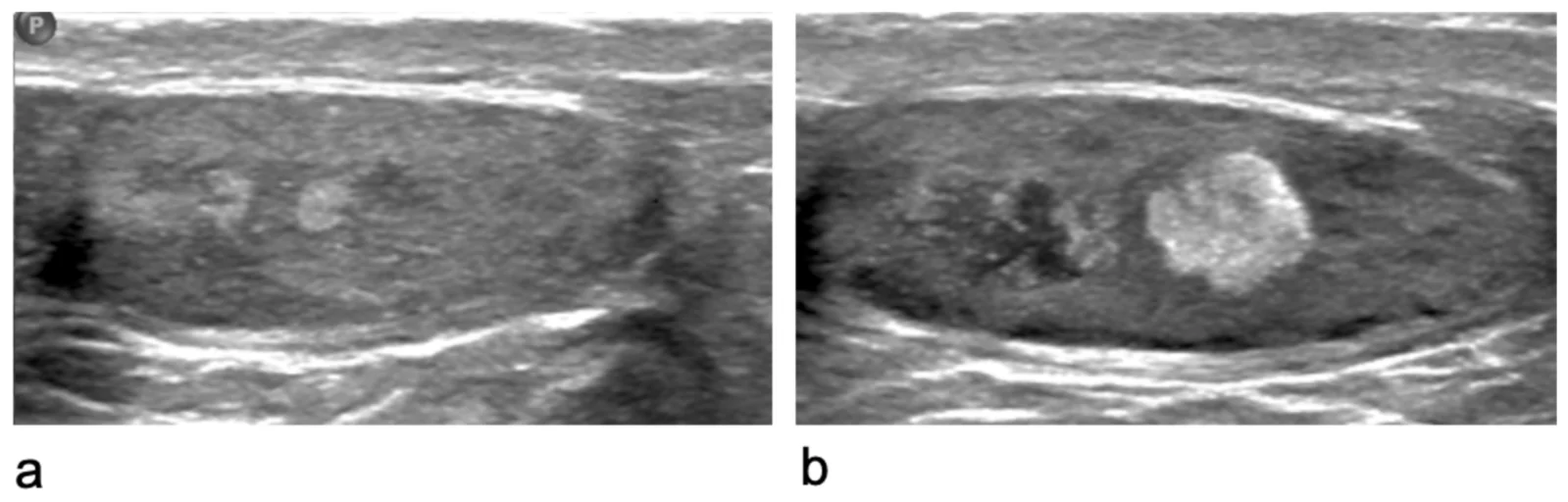
A 26-year-old man was referred for ultrasound evaluation because of azoospermia and markedly hypotrophic testes. The right (**a**) and left (**b**) testes were small, with heterogeneous echotexture and multiple non-palpable lesions of variable echogenicity. The diagnosis of Klinefelter syndrome was confirmed by karyotyping.

Similarly, in Peutz–Jeghers syndrome and Carney complex, large-cell calcifying Sertoli cell tumours may present as bilateral or multifocal lesions, frequently with calcifications, and usually have low malignant potential [74,75]. In Klinefelter syndrome, heterogeneous or coarse micronodular parenchymal change, with multiple small nodules that are usually hypoechoic but may occasionally show variable echogenicity, may reflect Leydig cell hyperplasia or benign Leydig cell proliferations rather than malignancy (Figure 15) [9,73].

**Figure 16:**
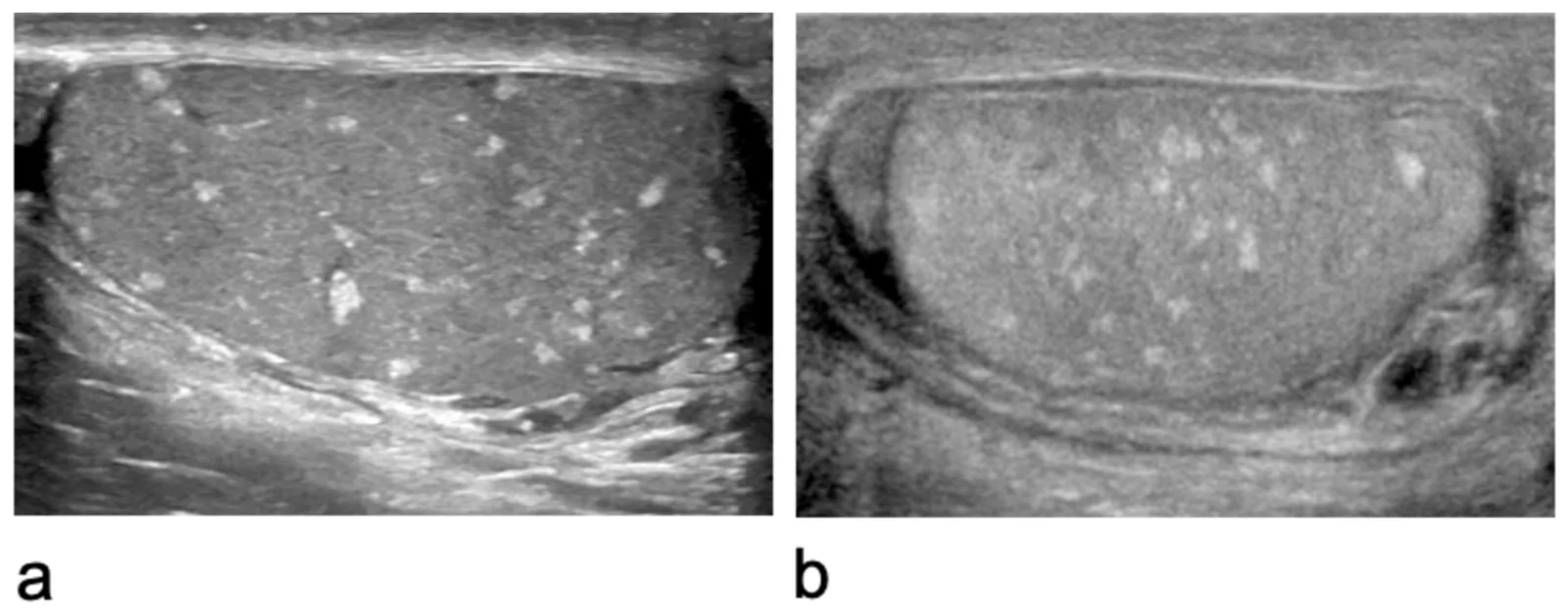
Two different patients (**a**,**b**) underwent scrotal ultrasound for unrelated indications. In both cases, ultrasound showed multiple bilateral hyperechoic intratesticular lesions. The diagnosis of Cowden syndrome was confirmed by diagnostic testing in patient (**a**), whereas testing was negative in patient (**b**).

A further important association is Cowden syndrome, in which multiple bilateral hyperechoic intratesticular lesions are classically related to testicular lipomatosis [76,77]. However, this appearance should not be regarded as pathognomonic, since hyperechoic intratesticular lesions, especially when solitary or atypical, may also reflect other benign non-neoplastic processes, including focal fibrosis, and therefore still require appropriate clinical and imaging correlation (Figure 16) [76,78].

In these syndromic settings, localisation, bilaterality and multiplicity are often more informative than lesion size alone. Conventional ultrasound remains the cornerstone of detection, while colour Doppler and microvascular imaging may improve the demonstration of vascularity in small lesions [9]. CEUS can further characterise lesion perfusion and help distinguish vascularised from truly avascular abnormalities, although enhancement patterns alone are not specific enough to differentiate benign syndrome-related lesions from neoplasia [9,15,51].

## 13. Lesion Detection in the Postoperative Scrotum

**Figure 17:**
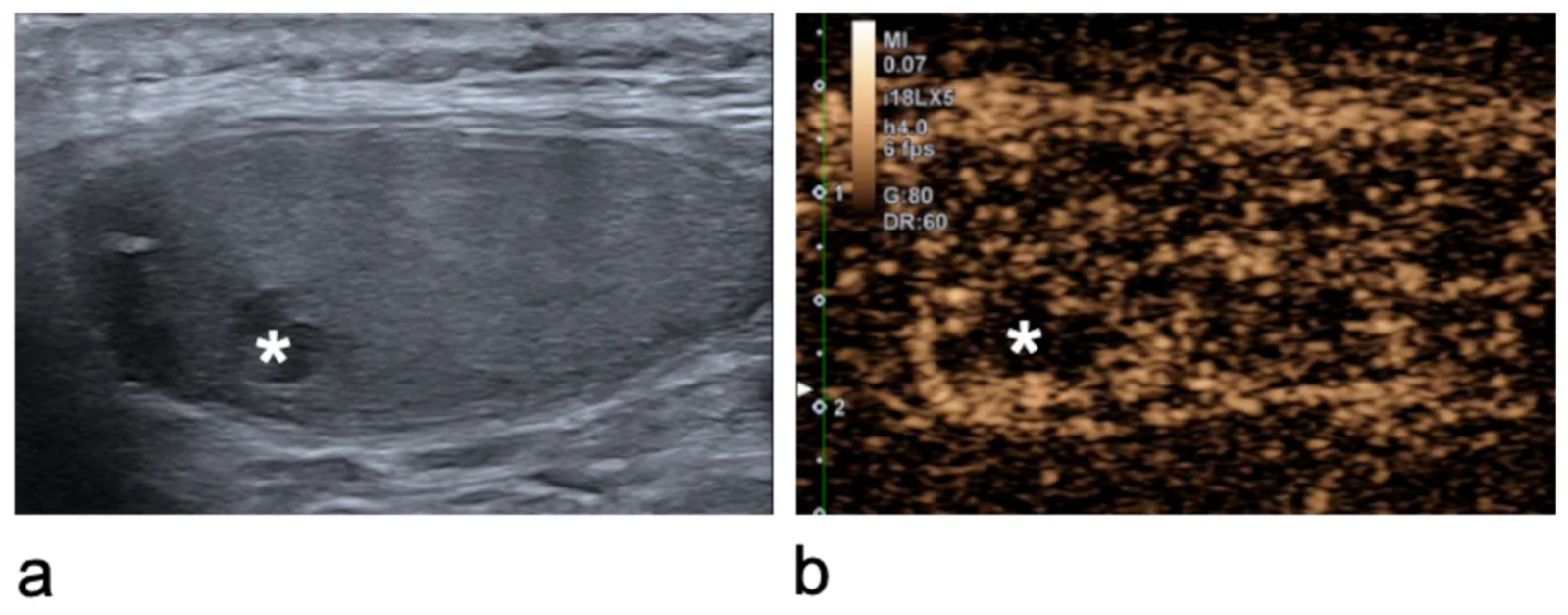
Ultrasound examination performed 40 h after testis-sparing surgery. (**a**,**b**) Ultrasound shows a parenchymal haematoma at the site of lesion excision (asterisk). The absence of vascularity of the lesion (asterisk) on CEUS allows differentiation from residual tumour.

With the increasing use of testis-sparing surgery (TSS) for selected focal testicular lesions, and of sperm-retrieval procedures in infertile men, imaging of the operated testis has become an important part of clinical practice. The postoperative scrotum is a challenging setting for ultrasound, because focal intratesticular abnormalities may represent expected postoperative or post-procedural changes rather than persistent, recurrent, or metachronous tumours. After TSS for focal lesions, however, the possibility of persistent or recurrent disease must always be considered [79,80,81]. Early after surgery, grey-scale ultrasound may show parenchymal distortion, heterogeneous echotexture, focal hypoechoic or mixed echogenic areas, small haematomas, and irregularity of the tunica or surgical bed. These abnormalities may be rounded or oval and can therefore mimic a neoplastic lesion (Figure 17) [63,82,83,84].

Ultrasound usually allows confident identification of tunica albuginea interruption, while CEUS may further delineate disruption of the tunica vasculosa and define adjacent non-viable or poorly perfused tissue. With time, benign postoperative change typically evolves towards focal scarring, albugineal thickening, contour deformity and, in some cases, testicular atrophy [82]. Established scars are commonly hypoechoic and hypovascular, often with a linear or roughly triangular configuration, rectilinear margins, and angular contours. Interval reduction in size or complete resolution supports a benign postoperative nature, whereas a persistent or enlarging rounded vascularised nodule should raise suspicion for recurrence [82]. Similar appearances may follow sperm-retrieval procedures in infertile patients [82,83,84]. In this context, vascular assessment is central. Postsurgical haematomas and scars are avascular or only perilesionally vascularised, whereas recurrent, persistent, or metachronous tumours are expected to show internal vascularity, particularly on microvascular imaging and CEUS [63,82]. Serial follow-up is also essential: post-procedural lesions usually become smaller and less conspicuous, whereas recurrence typically persists, enlarges, or develops internal vascularity, often adjacent to the surgical scar [63,82,83,84].

## 14. Conclusions

There is growing acceptance in contemporary urological practice that active surveillance is a reasonable option for incidentally detected, non-palpable testicular lesions ≤ 0.5 cm in diameter with negative tumour markers, whereas larger lesions are generally considered for surgical exploration [27,34,35]. During surveillance, lesions that remain stable can continue to be monitored, thereby avoiding unnecessary surgery in a substantial proportion of patients [27,34,43,85,86]. By contrast, lesions that enlarge should undergo surgery; in such cases, testis-sparing surgery is generally appropriate when growth is limited and imaging findings remain otherwise low-risk, whereas radical orchiectomy may be reserved for lesions that show faster interval growth and/or progressively more suspicious imaging findings during follow-up, particularly increasing intralesional vascularity and, where available, other concerning multiparametric ultrasound changes [4,87]. In appropriately selected patients, this strategy appears clinically sound: it reduces overtreatment by identifying many lesions that never require intervention and, among those that eventually require surgery, helps tailor the most appropriate approach, either conservative or radical, without apparent detriment to oncological outcome [34,43,85,86]. However, current urological guidelines remain more conservative, and surgery, most commonly orchiectomy, is still generally recommended. The rationale is that even very small testicular incidentalomas may harbour malignancy in a minority of cases: a systematic review pooling six studies and 293 patients managed with radiological surveillance reported malignancy in 3.4% of cases (10/293; 95% CI 1.9–6.2%) [85], while a single-centre retrospective cohort of 161 men managed with surveillance reported a malignancy rate of 4.3% (7/161; 95% CI 2.1–8.7%) [86]; delaying treatment might theoretically worsen prognosis. This concern is partly based on the observation that primary tumour size is a predictor of relapse in clinical stage I seminoma. Nevertheless, the available prognostic data derive largely from orchidectomy series involving tumours measuring around 3–4 cm, rather than from incidental subcentimetric lesions managed with short-interval surveillance [88,89]. By contrast, recent surveillance cohorts of subcentimetric, non-palpable, marker-negative lesions suggest that malignant tumours identified after interval growth are still usually detected at a localised stage [43]. More importantly, prognosis in testicular germ-cell tumours is driven primarily by tumour biology and disease stage, rather than by millimetric size increase alone. Current staging systems do not indicate that a lesion initially compatible with stage I disease, which enlarges during follow-up but is still treated while remaining below 1–2 cm and confined to the testis, necessarily carries a worse prognosis solely because of that increase in size. Thus, in small testicular incidentalomas, the key issue is not minor dimensional progression per se, but whether surveillance allows intervention before biologically meaningful progression or stage migration occurs. Current evidence suggests that, within a structured follow-up programme, this is generally the case [34,43,85,86].

In this context, imaging has become central to clinical decision-making, improving lesion characterisation beyond conventional B-mode and colour Doppler imaging and supporting a more individualised approach. It cannot rely on any single clinical or imaging feature, but requires integration of imaging features with patient history, tumour markers, lesion size, grey-scale appearance, vascularity, stiffness, and temporal evolution.

Magnetic resonance imaging (MRI) has been advocated as a valuable adjunct for characterising sonographically indeterminate testicular lesions, and dedicated recommendations for its use in the scrotum have been published [90]. In our experience, however, its role in the routine characterisation of focal testicular lesions remains limited, notwithstanding some literature suggesting otherwise [91,92,93]. The principal added value of MRI lies in tissue characterisation, specifically the recognition of fat and haemorrhagic components, but this is relevant only to a minority of lesions. Assessment of the homogeneity or heterogeneity of signal intensity may help distinguish seminomatous from non-seminomatous germ cell tumours, although this is mainly applicable to larger lesions. MRI is therefore most useful in selected scenarios in which ultrasound assessment is technically difficult, such as markedly inhomogeneous testicular parenchyma or extensive, particularly dense testicular microlithiasis. For small lesions, the diagnostic contribution of MRI is limited, partly because of its lower spatial resolution compared with ultrasound. Although MRI has been reported to be more sensitive than colour Doppler ultrasound for depicting lesion vascularity, CEUS is generally preferable in clinical practice, being cheaper, more practical, and able to be performed immediately during the same ultrasound examination [94]. Similarly, while dynamic contrast-enhanced MRI enhancement curves have been proposed to differentiate benign from malignant testicular lesions, this has not been our experience: substantial overlap between curve types is observed, and reliance on this feature alone may be unsafe. Overall, ultrasound, supported where necessary by CEUS, remains the modality of choice for the initial assessment and follow-up of testicular lesions, with MRI reserved as a second-line, problem-solving tool in selected cases.

This approach may help reduce unnecessary orchidectomy, particularly in small incidentalomas, syndromic lesions, acute scrotal conditions, traumatic lesions, and the postoperative testis, while maintaining oncological vigilance for malignant, recurrent, synchronous, or metachronous disease.

Emerging radiomics and artificial intelligence-based analysis of multiparametric ultrasound and cross-sectional imaging [94] hold promise for further refining risk stratification, standardising image interpretation, and reducing inter-observer variability, representing a valuable future complement to conventional lesion characterisation.

## Data Availability

No new data were created or analysed in this study. Data sharing is not applicable to this article.

## Abbreviations

The following abbreviations are used in this manuscript:

TSS	Testis-sparing surgery
TGCTs	Testicular germ cell tumours
AFP	Alpha-fetoprotein
β-hCG	Beta-human chorionic gonadotropin
LDH	Lactate dehydrogenase
CEUS	Contrast-enhanced ultrasound
MVI	Microvascular imaging
CAH	Congenital adrenal hyperplasia
TARTs	Testicular adrenal rest tumours
MRI	Magnetic resonance imaging
